# CA125 effects on humoral immunosuppression

**DOI:** 10.18632/aging.101301

**Published:** 2017-10-03

**Authors:** J. Bradford Kline, Nicholas C. Nicolaides

**Affiliations:** Morphotek Inc., Exton, PA 19342, USA

**Keywords:** CA125, farletuzumab, humoral immunosuppression, ADCC, ovarian cancer

Cancer-mediated immune suppression is a growing field as the complex mechanisms tumors deploy to avoid host defense become better understood. While the majority of research efforts are focusing on the pathways affecting cellular immunosuppression, tumors employ a diverse array of other mechanisms to ensure their survival [1]. One such alternative mechanism is the ability to suppress humoral-based immunity. A variety of cancers are known to produce tumor-shed antigens (TSAs), i.e. CEA, CA125, CA15-3, AFP, etc., that enhance tumor cell survival and/or metastasis. These effects can have a significant impact on the efficacy of natural and/or therapeutic agents that utilize humoral-based immune mechanisms.

Ovarian cancer is a leading cause of gynecological cancer worldwide. In the U.S. alone, greater than 20,000 women will be diagnosed and more than 14,000 will die of the disease per year [2]. CA125 is a TSA commonly found in the serum of patients with serous epithelial ovarian cancer (EOC). It is overexpressed on tumor cell membranes in most EOCs and in subsets of other cancers (i.e., mesothelioma, lung, pancreatic and breast). It is a large glycoprotein (>22,000 amino acids) comprised of a heavily O-glycosylated N-terminal region, a tandem repeat region of approximately sixty 156 residue repeats, a transmembrane domain and a short cytoplasmic tail [3].

Previous studies show multiple roles for CA125 in tumor survival. Expression of the C-terminal domain has been shown to induce cellular transformation and tumor invasion [4]. Both soluble (sCA125) and membrane-bound CA125 (mCA125) forms have been shown to inhibit immune-effector activities of lymphocytes, in particular antibody-dependent cellular cytotoxicity (ADCC). These have been reported to occur by sCA125 binding to Siglec-type receptors and causing downregulation of Fc-γ activating receptors while mCA125 has exhibited immunosuppressive effects on NK cell-mediated ADCC by forming physical barriers that suppress this function [3].

Farletuzumab, is an investigational humanized IgG1-type monoclonal antibody (mAb) targeting folate receptor alpha (FRA), which is expressed on a high percentage of ovarian cancers. In preclinical models, it mediates ADCC in part to kill FRA-expressing tumors. Recently, an 1100 patient phase 3 clinical trial in patients with first-relapsed, platinum-sensitive EOC concluded (NCT00849667) [5]. While the study did not meet its primary endpoint, prespecified subgroup analysis identified a responding subpopulation (N = 290) whereby patients treated with carboplatin and taxane (CT) plus farletuzumab had improvements in progression-free and overall survival over those treated with CT and placebo (Hazard Ratio [HR] 0.49; p = 0.0028 and HR 0.44, p = 0.0108, respectively). Other TSAs showed no correlation [6]. Based on these observations, further characterization of the potential immunosuppressive effects of CA125 on farletuzumab were investigated.

*In vitro*, sCA125 and mCA125 both have profound immunosuppressive effects on farletuzumab-mediated ADCC by primary NK and PBMC-derived effector cells in a dose-dependent manner. Moreover, the effective concentrations of sCA125 were found to be at levels frequently observed in the tumor microenvironment.

ADCC of antibodies relies on the engagement of the antibody on target tumor cells that in turn bridge the tumor and NK/effector cell via Fc-γ activating receptors (refer to Figure 1 top panel). CD16a is the major Fc-γ activating receptor on NK cells. Independent studies have shown that engagement of CD16a with a cell surface-bound antibody results in NK cell activation and release of lytic granules that lead to target cell killing [6]. Inhibition of this interaction results in suppressed ADCC. Recently, we showed that CA125 can bind to farletuzumab and suppress its ability to physically engage with the CD16a receptor on effector cells as well as purified CD16a receptor [7]. Competition assays using farletuzumab full length or Fc and (Fab‘)_2_ fragments found that the immuno-suppressive effect of sCA125 could be inhibited using the (Fab‘)_2_ fragment, suggesting that CA125 inhibits Fc-γ receptor engagement by binding to a region within the (Fab‘)_2_ domain.

**Figure 1:**
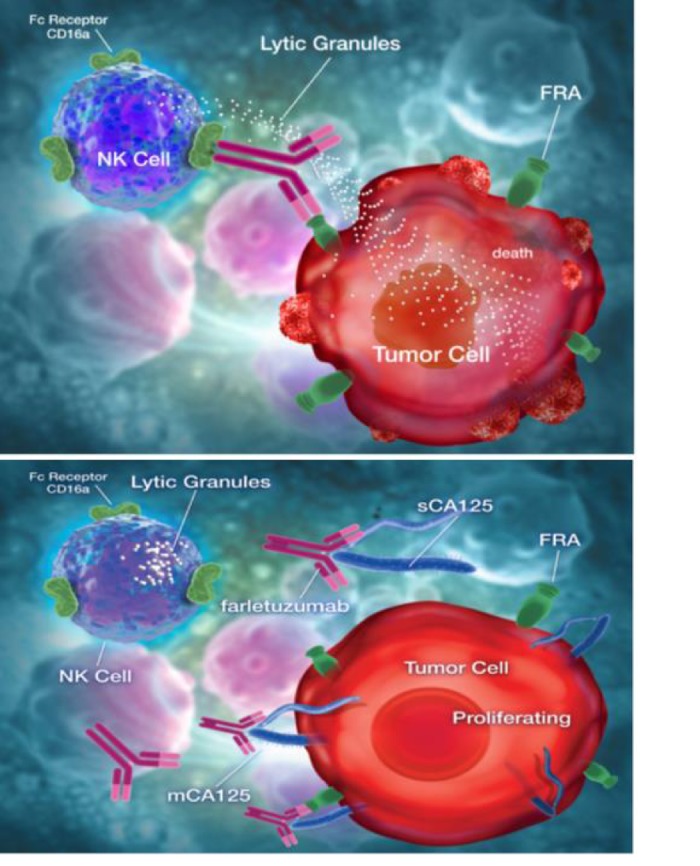
Model depicting farletuzumab ADCC by NK cells on FRA-expressing tumor cells (top) and ADCC suppression by direct binding of CA125 to antibody (bottom).

Analysis of CA125 binding to other commercial and experimental therapeutic antibodies found that CA125 can bind to a subset of other antibodies regardless of format, i.e. fully human, humanized or chimerized. As these antibodies all share similar constant region domains, it suggests the major contact points for CA125 likely reside within the variable domain. Studies to identify the CA125 and antibody binding motif(s) on are ongoing.

The finding that tumors utilize TSAs such as CA125 as an alternative mechanism for suppressing a patient's immune system offers new insights into the complex biological systems employed by cancers for survival. Additional understanding on how this effect occurs may offer new solutions to identifying people at risk for certain types of cancer, as well as new therapeutic strategies to improve clinical outcome. As part of this effort farletuzumab is currently in an ongoing clinical study in women with relapsed platinum-sensitive EOC exhibiting low CA125 levels (NCT02289950) to determine if improved clinical outcome can be achieved using humoral-based therapies in patients with low levels of immunosuppressive TSAs.

## References

[1] Hargadon KM (2013). Front Immunol.

[2] Siegel RL (2017). CA Cancer J Clin.

[3] Haridas D (2014). FASEB J.

[4] Rao TD (2015). PLoS One.

[5] Vergote I (2016). J Clin Oncol.

[6] Carter P (2001). Nat Rev Cancer.

[7] Kline JB (2017). Oncotarget.

